# Influence of weight concerns on breastfeeding: Evidence from the Norwegian mother and child cohort study

**DOI:** 10.1002/ajhb.23086

**Published:** 2017-11-27

**Authors:** Seung‐Yong Han, Alexandra A. Brewis

**Affiliations:** ^1^ Mayo Clinic/Arizona State University Obesity Solutions Tempe Arizona 85287; ^2^ School of Human Evolution and Social Change Arizona State University Tempe Arizona 85287

## Abstract

**Objectives:**

High body mass index (BMI) often predicts truncated breastfeeding, although why is unclear. We test a proposed mediating role of body concerns on breastfeeding initiation and child's age at weaning using longitudinal data for 55,522 mothers from the Norwegian Mother and Child Cohort Study (MoBa).

**Methods:**

A linear regression‐based mediation analysis with bootstrapping estimates the indirect effects of BMI on breastfeeding decisions (ever‐initiation of breastfeeding, child's age at weaning, and duration of any breastfeeding beyond six months) through the variables of concern around prepregnancy weight and weight gains due to pregnancy.

**Results:**

Contrary to prediction, Norwegian mothers with greater prepregnancy weight concerns had a higher likelihood of initiating breastfeeding. Concerns about weight gain during pregnancy, however, predicted earlier weaning. This relationship was the same for higher and lower BMI mothers.

**Conclusion:**

In this very large sample, body image affects some breastfeeding decisions. However, this effect is independent of mother's body size.

## 1 INTRODUCTION

Obesity has been posed as a predictor of lower likelihood of initiating and continuing to breastfeed (Amir & Donath, 2007; Anstey & Jevitt, 2011; Kachoria, Moreland, Cordero, & Oza‐Frank, 2015; Manios et al., 2009; Mäkelä, Vaarno, Kaljonen, Niinikoski, & Lagström, 2014; Turcksin, Bel, Galjaard, & Devlieger, 2014; Verret‐Chalifour et al., 2015). Reasons include difficult births and delayed lactogenesis II related to having high body fat (see Babendure, Reifsnider, Mendias, Moramarco, & Davila, 2015 for review). However, breastfeeding decisions are driven to a large extent by maternal intention and decision‐making. In this journal, Hauff and Demerath (2012) suggested and demonstrated a further mechanism: body issues. Following 257 primiparous women in Minnesota, USA, for six months postpartum, they found that women with high prepregnancy body mass index (BMI) had *reduced* breastfeeding duration, and this was seemingly mediated by women's body discomfort. Another study by Brown, Rance, and Warren (2015) followed 128 pregnant women in the U.K. for six months postpartum and found negative associations between three different types of body image concerns and breastfeeding duration. The main reported reasons for stopping breastfeeding were embarrassment regarding public feeding and the impact of breastfeeding on breast shape. A qualitative study of Keely, Lawton, Swanson, and Denison (2015) echoed this finding: lack of privacy for breastfeeding discouraged new mothers from continued breastfeeding. By contrast, a case‐control study by Zanardo et al. (2014) of 25 overweight/obese and 25 nonoverweight/obese Italian mothers found women with higher BMI had more body dissatisfaction, yet were more likely to still be breastfeeding at six months postpartum. However, analysis of longitudinal data for 2824 women in the Infant Feeding Practices Study II showed overweight/obese women had similar intention to breastfeed as peers, but were less likely to initiate breastfeeding, and terminated breastfeeding sooner (Hauff, Leonard, & Rasmussen, 2014).

Accordingly, evidence regarding the relationship between women's body issues and decisions around breastfeeding remains confusing. This study provides the means to test the proposal that body image/concerns play a significant and direct role in breastfeeding among women with high BMI with a very large, longitudinal sample from the Norwegian Mother and Child Cohort Study (MoBa).

## 2 DATA AND METHODS

The Norwegian Mother and Child Cohort Study (MoBa) is a prospective population‐based pregnancy cohort study conducted by the Norwegian Institute of Public Health. Participants were recruited across Norway from 1999 to 2008. The cohort now includes 114,500 children, 95,200 mothers, and 75,200 fathers. The current study is based on version 8 of the quality‐assured data files released for research on the breastfeeding behavior of Norwegian mothers (Magnus et al., 2016).

We modeled the data collected during pregnancy, and at 6 and 18 months postpartum. Our analytic sample was 55,522 mothers: 38,026 classified as nonoverweight/obese (18.5 ≤ BMI < 25) and 17,496 classified as overweight/obese (BMI ≥ 25). Those with BMI classified as underweight (<18.5) were excluded.

**Figure 1:**
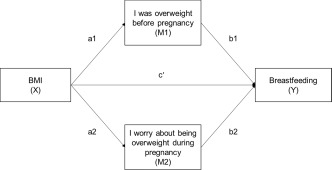
Mediation model of body mass index, weight concerns, and breastfeeding outcomes. Note: a user‐written *binary_mediation* command with bootstrapping option in Stata 14.2 was used to estimate indirect, direct, and total effects

Three breastfeeding outcomes were tested: ever‐initiation of breastfeeding (yes or no), total duration of breastfeeding (months), and whether breastfeeding extended beyond 6 months (yes or no). Body concerns were assessed by two interview questions: “*Do you think you were overweight just before this pregnancy?*” and “*Are you worried about putting on more weight than necessary during this pregnancy?*” Scores were based on responses of 0 = “*no*” or “*not especially worried*”, 1 = “y*es, a little*” or “s*omewhat worried*”, and 2 = “*yes, a lot*” or “*yes, very worried”*. We employed mediation analysis to test the direct influence of BMI on breastfeeding initiation and duration, and then the mediation effect provided by body concern variables (Figure 1). Analysis procedures included a *binary_mediation* command with 5000 bootstrapped samples in Stata 14.2. In detail, the effects of BMI on (1) prepregnancy weight concern (path a1 in Figure 1), (2) concern about weight gain during pregnancy (path a2 in Figure 1), and (3) their effects on breastfeeding (path b1 and b2 in Figure 1) were estimated by using ordinary least square regression models. Then, whether or not each product of two estimated coefficients (path a1 × path b1 and path a2 × path b2) is significantly different from zero was tested at the *p*‐value .05 level.

## 3 RESULTS

The proportion of new mothers initiating breastfeeding was 82% for nonoverweight/obese women compared to 81% for overweight/obese women; this was statistically significant given the very large sample size (z statistic = 3.2620, *p*‐value = .0011). Average duration of breastfeeding was 9 months for nonoverweight/obese women, and 8 months for overweight/obese women, also a significant difference (*t* statistic = −13.8944, *p*‐value < .0001).

As shown in Table 1, the analysis confirmed that increasing maternal prepregnancy BMI predicted shorter duration of breastfeeding (total effect). Women with lower BMI were more likely to wean later. This association was stronger for overweight/obese women. However, for nonoverweight/obese women, the association was still positive when the initiation of any breastfeeding was considered as an outcome.

**Table 1:** Mediation analysis results of the association between BMI (X) and breastfeeding (Y1, Y2, Y3) with prepregnancy weight concern (M1) and pregnancy weight gain concern (M2) are mediators

		Overweight/Obese(25 ≤ BMI)		Healthy(18.5 ≤ BMI < 25)	
Effects	Paths	Coef.	95% C.I.^a^	Coef.	95% C.I.^a^
*Y1: Breastfeeding initiation (1/0)*					
Total indirect effect		**0.013**	(0.000; 0.025)	**0.007**	(0.000; 0.015)
*Indirect effect 1*	(BMI → weight concern → initiation)	**0.016**	(0.003; 0.028)	**0.010**	(0.003; 0.018)
*Indirect effect 2*	(BMI → weight gain concern → initiation)	−0.003	(–0.007; 0.002)	–**0.003**	(–0.005; −0.001)
Direct effect	(BMI (weight & weight gain concern) → initiation)	−0.007	(–0.033; 0.019)	**0.021**	(0.005; 0.037)
Total effect	(BMI → initiation)	0.006	(–0.016; 0.028)	**0.029**	(0.015; 0.043)
*Y2: Breastfeeding duration (months)*					
Total indirect effect		0.007	(–0.001; 0.015)	−0.001	(–0.006; 0.004)
*Indirect effect 1*	(BMI → weight concern → duration)	**0.014**	(0.006; 0.023)	0.003	(–0.002; 0.009)
*Indirect effect 2*	(BMI → weight gain concern → duration)	–**0.007**	(–0.010; −0.004)	–**0.005**	(–0.007; −0.004)
Direct effect	(BMI (weight & weight gain concern) → duration)	–**0.066**	(–0.082; −0.049)	−0.006	(–0.018; 0.006)
Total effect	(BMI → duration)	–**0.059**	(–0.073; −0.044)	−0.008	(–0.018; 0.003)
*Y3: Breastfeeding duration over 6 months (1/0)*					
Total indirect effect		**0.010**	(0.001; 0.020)	−0.003	(–0.009; 0.002)
*Indirect effect 1*	(BMI → weight concern → Over 6)	**0.016**	(0.006; 0.026)	0.002	(–0.004; 0.008)
*Indirect effect 2*	(BMI → weight gain concern → Over 6)	–**0.005**	(–0.009; −0.002)	–**0.005**	(–0.007; −0.003)
Direct effect	(BMI (weight & weight gain concern) → Over 6)	–**0.097**	(–0.117; −0.077)	−0.001	(–0.015; 0.012)
Total effect	(BMI → Over 6)	–**0.087**	(–0.104; −0.069)	−0.005	(–0.017; 0.007)

Note: Bootstrapping results after 5000 successful replications; **Bold** if the result is significant at least at the .05 level of significance; self‐esteem, social support, stress, exercise, general health, education, income, immigration status, pregnant before, and age are controlled for in all models; ^a^ bias corrected 95% confidence interval; See Appendix 1, 2, 3, 4, and 5 for full results; sample sizes: 38,026 (nonoverweight/obese) and 17,496 (overweight/obese).

The mediation results (indirect effect 1 and 2 in Table 1) show that both body image variables (prepregnancy weight concern and pregnancy weight gain concern) significantly mediated the association between BMI and any breastfeeding, but in different directions. For women in all BMI categories, prepregnancy weight concern (M1) showed a significant mediation effect (indirect effect 1). In detail, one unit increase in BMI was associated with 1.6% (overweight or obese) and 1.0% (healthy) increase in the likelihood of breast feeding initiation via prepregnancy weight concern, respectively. That is, women who had higher BMI were more concerned about their weight at the start of their pregnancies, and they were more likely to breastfeed at all. For overweight/obese women only, prepregnancy weight concern (M1) also mediated the total duration of any breastfeeding (.02 months increase per 1 kg/m^2^) and the likelihood of breastfeeding more than 6 months (1.6% increase in the likelihood per 1 kg/m^2^). On the other hand, women concerned about weight gain during pregnancy (M2) truncated the duration of any breastfeeding significantly. They also had a reduced likelihood of longer‐term breastfeeding (>6 months) (indirect effect 2), but the effect was small (.05% decrease per 1 kg/m^2^). The results of the covariates controlled for in the model were consistent with those observed in prior studies.

## 4 DISCUSSION

The results confirm that Norwegian mothers with greater concern about their weight *before* pregnancy had better breastfeeding outcomes. That is, they were more likely to initiate breastfeeding and to wean later. For initiation of breastfeeding, this effect was observed in both higher and lower BMI women. Overweight/obese mothers, on average, weaned sooner. But body issues did not mediate this relationship. Overweight/obese mothers with greater prepregnancy weight concerns breastfeed for *longer*.

Concerns about weight gain *due to* pregnancy predicted worse breastfeeding outcomes, but these were not related to women's BMI. Women in all weight categories with weight concerns around pregnancy weaned sooner. These results are not fully consistent with what has been observed in prior studies, and do not support the proposition that high body weight shapes worse breastfeeding outcomes through the mechanism of body weight concerns.

We can make several suggestions as to why our results somewhat contradict prior studies. First, MoBa provides a very large sample with high statistical power, and prospectively tracks women through time. A high statistical power allowed us to detect a relatively small mediation effect for continuous breastfeeding duration as well as meaningful size of the medication effects of prepregnancy weight concern on initiation and long‐term breastfeeding in our study. Prior studies had solid design (case control, longitudinal), but much smaller samples. It may be that the observed effects disappeared with much higher statistical power.

Second, Norwegian women might be different than other populations of women, such that weight issues may be less salient for overweight/obese Norwegian women compared to women included in previous studies. We think, however, this is unlikely. Norwegians, compared to other groups, exhibit very high levels of anti‐fat sentiment (Malterud & Ulriksen, 2010). It could be that, accordingly, Norwegian women with weight concerns are deploying extended breastfeeding as a weight loss strategy, something that cannot be identified in the available dataset.

It could also be that the questions used regarding weight concerns in the MoBa are unable to adequately capture relevant constructs of body image concern. For example, the Hauff and Demereth study collected concerns about specific body shape, not just weight, and specifically questioned women about their body concerns while breastfeeding in public.

This study, together with prior studies with smaller samples, suggests that body issues impact breastfeeding choice and outcomes in women of different body sizes in possibly very complex, situationally structured ways. More detailed study will be required to sort through the underlying mechanisms.

## Supporting information

Additional Supporting Information may be found online in the supporting information tab for this article.

Supporting Information
