# Supplementary material for: Influence of weight concerns on breastfeeding: Evidence from the Norwegian mother and child cohort study
Source: Am J Hum Biol. 2017 Nov 27;30(2):e23086. doi: 10.1002/ajhb.23086 (PMC5947548; doi:10.1002/ajhb.23086)
Supplement: Supplementary file 1 — Supporting Information [file AJHB-30-na-s001.docx]

| <Appendix 1> Summary Statistics of Breastfeeding Measures, BMI, Mediators, and Covariates by Weight Status | | | | | | |
| --- | --- | --- | --- | --- | --- | --- |
|  | Overweight / Obese  (25≤BMI) | | | Healthy  (18.5≤BMI<25) | | |
|  | Mean (S.D.)  or % | Min | Max | Mean (S.D.)  or % | Min | Max |
| *Main variables* |  |  |  |  |  |  |
| Breastfeeding |  |  |  |  |  |  |
| *Initiation (Y1)* | 82% | 0 | 1 | 81% | 0 | 1 |
| *Duration (Y2)* | 8.29 (6.20) | 0 | 17.5 | 9.08 (6.22) | 0 | 17.5 |
| *Over 6 months (Y3)* | 61% | 0 | 1 | 68% | 0 | 1 |
| BMI before pregnancy (X) | 28.94 (3.74) | 25 | 59.5 | 21.96 (1.67) | 18.5 | 25 |
| Weight concern (M1) | 1.26 (0.60) | 0 | 2 | 0.33 (0.50) | 0 | 2 |
| Weight gain concern (M2) | 0.73 (0.71) | 0 | 2 | 0.39 (0.59) | 0 | 2 |
|  |  |  |  |  |  |  |
| *Covariates* |  |  |  |  |  |  |
| Age | 30.52 (4.47) | 18 | 47 | 30.43 (4.26) | 18 | 47 |
| Self-esteem | 9.15 (1.97) | 0 | 12 | 9.41 (1.88) | 0 | 12 |
| Exercise | 2.26 (1.46) | 0 | 5 | 2.26 (1.45) | 0 | 5 |
| Health | 1.14 (1.29) | 0 | 10 | 0.97 (1.17) | 0 | 10 |
| Relationship with a partner | 21.25 (3.44) | 0 | 25 | 21.34 (3.40) | 0 | 25 |
| Relationship with a family |  |  |  |  |  |  |
| *Talk rarely* | 1% | 0 | 1 | 1% | 0 | 1 |
| *Talk occasionally* | 19% | 0 | 1 | 21% | 0 | 1 |
| *Talk often* | 80% | 0 | 1 | 78% | 0 | 1 |
| Stress | 88% | 0 | 1 | 85% | 0 | 1 |
| Higher education | 60% | 0 | 1 | 71% | 0 | 1 |
| Household income |  |  |  |  |  |  |
| *Low income* | 11% | 0 | 1 | 10% | 0 | 1 |
| *Mid income* | 80% | 0 | 1 | 75% | 0 | 1 |
| *High income* | 10% | 0 | 1 | 15% | 0 | 1 |
| Foreign-born | 8% | 0 | 1 | 11% | 0 | 1 |
| Pregnant before | 69% | 0 | 1 | 64% | 0 | 1 |
| N | 17,496 | | | 38,026 | | |

| <Appendix 2> Ordinary Least Square Regression Estimates of the Associations between BMI (X) and Mediators (M1 and M2) | | | | | | | | |
| --- | --- | --- | --- | --- | --- | --- | --- | --- |
|  | On Pre-pregnancy weight concern (M1) | | | | On Pregnancy weight gain concern (M2) | | | |
|  | Overweight / Obese  (25≤BMI) | | Healthy  (18.5≤BMI<25) | | Overweight / Obese  (25≤BMI) | | Healthy  (18.5≤BMI<25) | |
|  | Coef. (S.E.) |  | Coef. (S.E.) |  | Coef. (S.E.) |  | Coef. (S.E.) |  |
| BMI (X) | 0.08 (0.00) | ^***^ | 0.13 (0.00) | ^***^ | 0.03 (0.00) | ^***^ | 0.05 (0.00) | ^***^ |
| Age | 0.00 (0.00) | ^***^ | 0.00 (0.00) | ^†^ | 0.00 (0.00) | ^**^ | -0.01 (0.00) | ^***^ |
| Self-esteem | -0.04 (0.00) | ^***^ | -0.04 (0.00) | ^***^ | -0.07 (0.00) | ^***^ | -0.05 (0.00) | ^***^ |
| Exercise | -0.01 (0.00) | ^*^ | 0.00 (0.00) | ^**^ | 0.02 (0.00) | ^***^ | 0.03 (0.00) | ^***^ |
| Health | 0.01 (0.00) | ^***^ | 0.02 (0.00) | ^***^ | 0.01 (0.00) | ^**^ | 0.02 (0.00) | ^***^ |
| Relationship with a partner | 0.00 (0.00) | ^***^ | -0.01 (0.00) | ^***^ | -0.01 (0.00) | ^***^ | 0.00 (0.00) | ^***^ |
| Relationship with a family |  |  |  |  |  |  |  |  |
| *Talk rarely* | -0.06 (0.04) |  | 0.02 (0.03) |  | -0.04 (0.06) |  | 0.04 (0.03) |  |
| *Talk occasionally* | -0.02 (0.01) | ^†^ | -0.01 (0.01) |  | -0.04 (0.01) | ^**^ | -0.03 (0.01) | ^***^ |
| Stress | 0.05 (0.01) | ^***^ | 0.02 (0.01) | ^**^ | 0.10 (0.02) | ^***^ | 0.06 (0.01) | ^***^ |
| Higher education | 0.04 (0.01) | ^***^ | -0.01 (0.01) | ^**^ | 0.01 (0.01) |  | -0.03 (0.01) | ^***^ |
| Household income |  |  |  |  |  |  |  |  |
| *Low income* | -0.03 (0.01) | ^*^ | 0.00 (0.01) |  | -0.09 (0.02) | ^***^ | -0.01 (0.01) |  |
| *High income* | 0.02 (0.01) | ^†^ | 0.04 (0.01) | ^***^ | 0.08 (0.02) | ^***^ | 0.08 (0.01) | ^***^ |
| Foreign-born | 0.05 (0.01) | ^**^ | 0.03 (0.01) | ^*^ | 0.06 (0.02) | ^**^ | 0.07 (0.01) | ^***^ |
| Pregnant before | 0.07 (0.01) | ^***^ | 0.05 (0.01) | ^***^ | -0.03 (0.01) | ^**^ | -0.06 (0.01) | ^***^ |
| Intercept | -0.72 (0.05) | ^***^ | -2.05 (0.04) | ^***^ | 0.47 (0.07) | ^***^ | 0.10 (0.05) | ^†^ |
| F test | 463.16^***^ | | 775.06^***^ | | 114.86^***^ | | 203.31^***^ | |
| Adjusted R-square | .27 | | .22 | | .08 | | .07 | |
| N | 17,496 | | 38,026 | | 17,496 | | 38,026 | |
| Note: : ^†^ p<.10; ^*^ *p* <.05; ^**^ *p* <.01; ^***^ *p* <.001, two tailed; | | | | | | | | |

| <Appendix 3> Logistic Regression Estimates of Direct and Total Effects of BMI on Breastfeeding Initiation (Y1) | | | | | | | | |
| --- | --- | --- | --- | --- | --- | --- | --- | --- |
|  | Overweight / Obese  (25≤BMI) | | | | Healthy  (18.5≤BMI<25) | | | |
|  | Model 1  (total effect) | | Model 2  (direct effect) | | Model 1  (total effect) | | Model 2  (direct effect) | |
|  | Coef. (S.E.) |  | Coef. (S.E.) |  | Coef. (S.E.) |  | Coef. (S.E.) |  |
| Weight concern (M1) |  |  | 0.10 (0.04) | ^*^ |  |  | 0.09 (0.03) | ^**^ |
| Weight gain concern (M2) |  |  | -0.04 (0.03) |  |  |  | -0.07 (0.02) | ^**^ |
| BMI (X) | 0.00 (0.01) |  | 0.00 (0.01) |  | 0.03 (0.01) | ^***^ | 0.02 (0.01) | ^**^ |
| Age | 0.01 (0.01) |  | 0.01 (0.01) |  | 0.02 (0.00) | ^***^ | 0.02 (0.00) | ^***^ |
| Self-esteem | 0.01 (0.01) |  | 0.01 (0.01) |  | 0.00 (0.01) |  | 0.00 (0.01) |  |
| Exercise | 0.00 (0.01) |  | 0.00 (0.01) |  | 0.02 (0.01) | ^*^ | 0.02 (0.01) | ^*^ |
| Health | 0.05 (0.02) | ^**^ | 0.05 (0.02) | ^**^ | 0.06 (0.01) | ^***^ | 0.06 (0.01) | ^***^ |
| Relationship with a partner | -0.03 (0.01) | ^***^ | -0.03 (0.01) | ^***^ | -0.02 (0.00) | ^***^ | -0.02 (0.00) | ^***^ |
| Relationship with a family |  |  |  |  |  |  |  |  |
| *Talk rarely* | -0.41 (0.19) | ^*^ | -0.40 (0.19) | ^*^ | -0.14 (0.15) |  | -0.13 (0.15) |  |
| *Talk occasionally* | 0.09 (0.05) |  | 0.09 (0.05) |  | 0.14 (0.03) | ^***^ | 0.13 (0.03) | ^***^ |
| Stress | 0.03 (0.06) |  | 0.03 (0.06) |  | 0.02 (0.04) |  | 0.02 (0.04) |  |
| Higher education | 0.49 (0.04) | ^***^ | 0.49 (0.04) | ^***^ | 0.34 (0.03) | ^***^ | 0.34 (0.03) | ^***^ |
| Household income |  |  |  |  |  |  |  |  |
| *Low income* | 0.06 (0.07) |  | 0.06 (0.07) |  | 0.08 (0.05) | ^†^ | 0.08 (0.05) |  |
| *High income* | 0.19 (0.08) | ^*^ | 0.19 (0.08) | ^*^ | 0.02 (0.04) |  | 0.02 (0.04) |  |
| Foreign-born | -0.02 (0.08) |  | -0.03 (0.08) |  | 0.04 (0.04) |  | 0.05 (0.04) |  |
| Pregnant before | -0.38 (0.05) | ^***^ | -0.38 (0.05) | ^***^ | -0.29 (0.03) | ^***^ | -0.30 (0.03) | ^***^ |
| Intercept | 1.68 (0.27) | ^***^ | 1.77 (0.27) | ^***^ | 0.39 (0.23) | ^†^ | 0.58 (0.23) | ^*^ |
| Likelihood Ratio test | 280.27^***^ | | 287.05^***^ | | 370.01^***^ | | 383.91^***^ | |
| Adjusted R-square | .02 | | .02 | | .01 | | .01 | |
| N | 17,496 | | 17,496 | | 38,026 | | 38,026 | |
| Note: : ^†^ p<.10; ^*^ *p* <.05; ^**^ *p* <.01; ^***^ *p* <.001, two tailed; | | | | | | | | |

| <Appendix 4> Ordinary Least Square Regression Estimates of Direct and Total Effects of BMI on Breastfeeding Duration (Y2) | | | | | | | | |
| --- | --- | --- | --- | --- | --- | --- | --- | --- |
|  | Overweight / Obese  (25≤BMI) | | | | Healthy  (18.5≤BMI<25) | | | |
|  | Model 1  (total effect) | | Model 2  (direct effect) | | Model 1  (total effect) | | Model 2  (direct effect) | |
|  | Coef. (S.E.) |  | Coef. (S.E.) |  | Coef. (S.E.) |  | Coef. (S.E.) |  |
| Weight concern (M1) |  |  | 0.30 (0.09) | ^**^ |  |  | 0.10 (0.07) |  |
| Weight gain concern (M2) |  |  | -0.33 (0.07) | ^***^ |  |  | -0.38 (0.06) | ^***^ |
| BMI (X) | -0.10 (0.01) | ^***^ | -0.11 (0.01) | ^***^ | -0.03 (0.02) |  | -0.02 (0.02) |  |
| Age | 0.11 (0.01) | ^***^ | 0.11 (0.01) | ^***^ | 0.14 (0.01) | ^***^ | 0.13 (0.01) | ^***^ |
| Self-esteem | 0.09 (0.03) | ^**^ | 0.08 (0.03) | ^**^ | 0.08 (0.02) | ^***^ | 0.07 (0.02) | ^***^ |
| Exercise | 0.06 (0.03) | ^*^ | 0.07 (0.03) | ^*^ | 0.14 (0.02) | ^***^ | 0.15 (0.02) | ^***^ |
| Health | 0.05 (0.04) |  | 0.05 (0.04) |  | 0.09 (0.03) | ^**^ | 0.09 (0.03) | ^**^ |
| Relationship with a partner | -0.09 (0.01) | ^***^ | -0.09 (0.01) | ^***^ | -0.06 (0.01) | ^***^ | -0.07 (0.01) | ^***^ |
| Relationship with a family |  |  |  |  |  |  |  |  |
| *Talk rarely* | -0.61 (0.50) |  | -0.61 (0.50) |  | -0.41 (0.37) |  | -0.40 (0.37) |  |
| *Talk occasionally* | 0.23 (0.12) | ^†^ | 0.22 (0.12) | ^†^ | 0.35 (0.08) | ^***^ | 0.34 (0.08) | ^***^ |
| Stress | -0.24 (0.14) | ^†^ | -0.22 (0.14) |  | -0.16 (0.09) | ^†^ | -0.13 (0.09) |  |
| Higher education | 1.99 (0.10) | ^***^ | 1.98 (0.10) | ^***^ | 1.80 (0.07) | ^***^ | 1.79 (0.07) | ^***^ |
| Household income |  |  |  |  |  |  |  |  |
| *Low income* | 0.29 (0.16) | ^†^ | 0.27 (0.16) | ^+^ | 0.47 (0.11) | ^***^ | 0.46 (0.11) | ^***^ |
| *High income* | 0.59 (0.16) | ^***^ | 0.61 (0.16) | ^***^ | -0.03 (0.09) |  | -0.01 (0.09) |  |
| Foreign-born | -0.05 (0.17) |  | -0.05 (0.17) |  | 0.18 (0.10) | ^†^ | 0.20 (0.10) | ^†^ |
| Pregnant before | -0.11 (0.11) |  | -0.14 (0.11) |  | -0.16 (0.07) | ^*^ | -0.19 (0.07) | ^**^ |
| Intercept | 7.73 (0.61) | ^***^ | 8.10 (0.62) | ^***^ | 4.61 (0.54) | ^***^ | 4.85 (0.56) | ^***^ |
| F test | 64.40^***^ | | 58.08^***^ | | 93.64^***^ | | 84.89^***^ | |
| Adjusted R-square | .05 | | .05 | | .03 | | .03 | |
| N | 17,496 | | 17,496 | | 38,026 | | 38,026 | |
| Note: : ^†^ p<.10; ^*^ *p* <.05; ^**^ *p* <.01; ^***^ *p* <.001, two tailed; | | | | | | | | |

| <Appendix 5> Logistic Regression Estimates of Direct and Total Effects of BMI on Breastfeeding Over 6 Months (Y2) | | | | | | | | |
| --- | --- | --- | --- | --- | --- | --- | --- | --- |
|  | Overweight / Obese  (25≤BMI) | | | | Healthy  (18.5≤BMI<25) | | | |
|  | Model 1  (total effect) | | Model 2  (direct effect) | | Model 1  (total effect) | | Model 2  (direct effect) | |
|  | Coef. (S.E.) |  | Coef. (S.E.) |  | Coef. (S.E.) |  | Coef. (S.E.) |  |
| Weight concern (M1) |  |  | 0.10 (0.03) | ^**^ |  |  | 0.01 (0.03) |  |
| Weight gain concern (M2) |  |  | -0.07 (0.02) | ^**^ |  |  | -0.12 (0.02) | ^***^ |
| BMI (X) | -0.04 (0.00) | ^***^ | -0.05 (0.00) | ^***^ | -0.01 (0.01) |  | 0.00 (0.01) |  |
| Age | 0.02 (0.00) | ^***^ | 0.02 (0.00) | ^***^ | 0.02 (0.00) | ^***^ | 0.02 (0.00) | ^***^ |
| Self-esteem | 0.03 (0.01) | ^**^ | 0.03 (0.01) | ^**^ | 0.03 (0.01) | ^***^ | 0.02 (0.01) | ^**^ |
| Exercise | 0.01 (0.01) |  | 0.01 (0.01) |  | 0.03 (0.01) | ^***^ | 0.04 (0.01) | ^***^ |
| Health | 0.01 (0.01) |  | 0.01 (0.01) |  | 0.02 (0.01) | ^†^ | 0.02 (0.01) | ^†^ |
| Relationship with a partner | -0.02 (0.00) | ^***^ | -0.02 (0.01) | ^***^ | -0.02 (0.00) | ^***^ | -0.02 (0.00) | ^***^ |
| Relationship with a family |  |  |  |  |  |  |  |  |
| *Talk rarely* | -0.21 (0.17) |  | -0.21 (0.17) |  | -0.22 (0.13) | ^†^ | -0.21 (0.13) | ^†^ |
| *Talk occasionally* | 0.08 (0.04) | ^*^ | 0.08 (0.04) | ^†^ | 0.12 (0.03) | ^***^ | 0.12 (0.03) | ^***^ |
| Stress | -0.11 (0.05) | ^*^ | -0.11 (0.05) | ^*^ | 0.00 (0.03) |  | 0.01 (0.03) |  |
| Higher education | 0.57 (0.03) | ^***^ | 0.57 (0.03) | ^***^ | 0.57 (0.02) | ^***^ | 0.57 (0.02) | ^***^ |
| Household income |  |  |  |  |  |  |  |  |
| *Low income* | -0.02 (0.05) |  | -0.02 (0.05) |  | 0.03 (0.04) |  | 0.03 (0.04) |  |
| *High income* | 0.24 (0.06) | ^***^ | 0.25 (0.06) | ^***^ | 0.07 (0.03) | ^*^ | 0.08 (0.03) | ^*^ |
| Foreign-born | -0.04 (0.06) |  | -0.04 (0.06) |  | 0.05 (0.04) |  | 0.06 (0.04) | ^†^ |
| Pregnant before | 0.01 (0.04) |  | 0.00 (0.04) |  | -0.05 (0.03) | ^*^ | -0.06 (0.03) | ^*^ |
| Intercept | 1.08 (0.21) | ^***^ | 1.19 (0.21) | ^***^ | -0.16 (0.19) |  | -0.12 (0.20) |  |
| Likelihood Ratio test | 661.54^***^ | | 676.15^***^ | | 863.29^***^ | | 898.83^***^ | |
| Adjusted R-square | .03 | | .03 | | .02 | | .02 | |
| N | 17,496 | | 17,496 | | 38,026 | | 38,026 | |
| Note: : ^†^ p<.10; ^*^ *p* <.05; ^**^ *p* <.01; ^***^ *p* <.001, two tailed; | | | | | | | | |
